# Thiazolidinedione use and risk of Parkinson’s disease in patients with type 2 diabetes mellitus

**DOI:** 10.1038/s41531-022-00406-8

**Published:** 2022-10-21

**Authors:** Houyu Zhao, Lin Zhuo, Yexiang Sun, Peng Shen, Hongbo Lin, Siyan Zhan

**Affiliations:** 1 Department of Epidemiology and Biostatistics, School of Public Health, Peking University, Beijing, China; 2 Research Center of Clinical Epidemiology, Peking University Third Hospital, Beijing, China; 3 Yinzhou District Center for Disease Control and Prevention, Ningbo, China; 4 Center for Intelligent Public Health, Institute for Artificial Intelligence, Peking University, Beijing, China

**Keywords:** Risk factors, Medical research

## Abstract

The health burden of Parkinson’s disease (PD) and diabetes increases rapidly in China. However, no population-based study of the association between glucose lowering agents and PD incidence has been conducted in mainland Chinese population. Preclinical studies indicate that thiazolidinediones (TZDs) have neuroprotective effects against PD through stimulating peroxisome proliferator-activated receptor gamma. Nevertheless, debate remains in human studies. We assembled a retrospective cohort of type 2 diabetes mellitus (T2DM) patients who were new users of TZDs or alpha glucosidase inhibitors (AGIs) using the Yinzhou Regional Health Care Database. A Cox model with inverse probability of treatment weighting (IPTW) was applied to estimate the hazard ratio (HR) of PD incidence associated with the use of TZDs compared with AGIs. The final cohort included 12,704 new users of TZDs and 49,696 new users of AGIs. The incidence of PD was 135 per 100,000 person-years in TZD users and 203 per 100,000 person-years in the AGI group. An inverse association between use of TZDs and incidence of PD, with a HR of 0.74 (95% confidence interval, 0.59–0.92), was observed after adjusting for potential confounding using IPTW. The results of various subgroup analyses and sensitivity analyses were consistent with the findings of the primary analysis. Our results indicated that the use of TZD is associated with a decreased risk of PD incidence in a mainland Chinese population with T2DM. Given the heavy disease burden of PD and diabetes in China, these findings could provide some evidence for developing effective prevention and control measures to reduce the future incidence of PD in China.

## Introduction

Parkinson’s disease (PD) is the second most prevalent neurodegenerative disease, characterized by motor features such as bradykinesia, rigidity, tremors, and postural instability^1^. This disorder contributes millions of disability adjusted life-years to the global burden of disease and is associated with a variety of risk factors^1,2^, among which type 2 diabetes mellitus (T2DM) is found to increase the risk of PD in a quite number of clinical studies^3,4^. This appears to be an important finding in the context of a rapidly increasing disease burden of diabetes and PD^1,5^. As these two diseases share several overlapping mechanisms such as oxidative stress, aberrant protein accumulation, mitochondrial dysfunction, and chronic inflammation^3,4^, much attention has been paid to the potential relationship between glucose lowering agents and neurodegeneration and whether these drugs could have some effects in preventing or treating PD^3,6,7^.

Thiazolidinediones (TZDs) are a class of important second-line glucose lowering agents, improving glycemic control in T2DM patients by increasing insulin sensitivity^3,7^. A variety of preclinical studies have suggested a neuroprotective effect of TZDs in PD models^3,4^, motivating several epidemiological studies of the association between TZD use and risk of PD incidence. Nevertheless, results of these studies are conflicting due to methodology heterogeneity, population difference, and various follow-up periods^8,9^. Furthermore, most of these studies have been conducted in Western countries, while only several similar studies of the association between TZD use and PD risk were reported in the Asia, where more than 60% of the world’s population aged over 65 years live^10^. Although, China has the highest disease burden and fastest growth of T2DM and PD^1,5^, no study has been performed to assess the effects of TZD use on PD risk in a mainland Chinese population. To fill this gap, we conducted a retrospective cohort study using a well-established electronic healthcare database in China to evaluate the association between TZD use and risk of PD in patients with T2DM.

## Results

### Basic characteristics

**Fig. 1:**
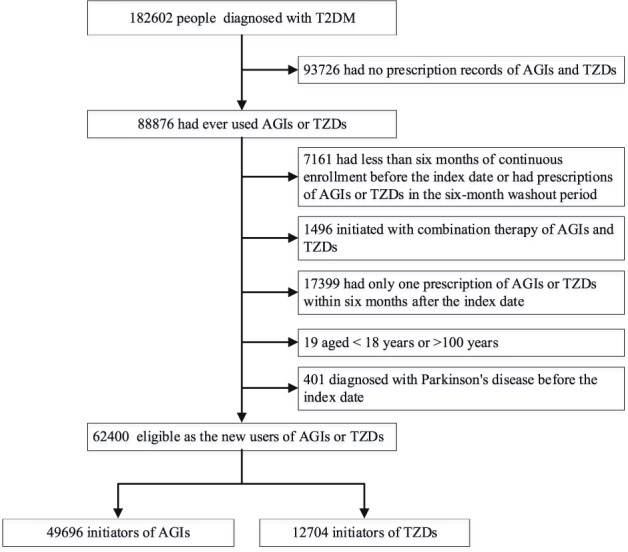
Flowchart of participants in the study cohort. T2DM type 2 diabetes mellitus, AGIs alpha glucosidase inhibitors, TZDs thiazolidinediones.

We included 62,400 T2DM patients in the final cohort. Of these, 49,696 participants initiated alpha glucosidase inhibitors (AGIs) and 12,704 were new users of TZDs (Fig. 1). The median follow-up time for new users of AGIs and TZDs was 5.3 (interquartile range [IQR], 2.5–8.7; maximum 13) and 6.1 (IQR, 3.1–8.8; maximum 13) years, respectively. Patients’ characteristics at baseline are given in the Table 1. Compared with AGI new users, there was a lower proportion of participants older than 60 years or having higher education in TZD users. Further, new users of TZDs were less likely to be hospitalized, have a Charlson comorbidity index (CCI) > 1, and receive insulin treatment in the baseline period. However, participants in the TZD group had higher body mass index (BMI) and were more likely to receive outpatient care and prescriptions of metformin and sulfonylureas at baseline. Nevertheless, after weighted by inverse probability of treatment, all baseline characteristics were effectively balanced between the exposure and comparison group (standardized mean difference [SMD] < 0.1 for all covariates, Table 1).

**Table 1:** Baseline covariates of new users of thiazolidinediones or alpha glucosidase inhibitors in the Yinzhou region^a^.

	Unweighted population			Weighted population		
	AGI users	TZD users	SMD	AGI users	TZD users	SMD
Age	61.1 (12.30)	59.0 (11.60)	0.175	60.7 (12.11)	60.5 (12.05)	0.043
Age >60 years	26,795 (53.9)	5992 (47.2)	0.135	26,131 (52.6)	6253 (50.7)	0.016
Years of T2DM	4.3 (5.03)	3.9 (4.22)	0.087	4.2 (4.85)	4.3 (4.85)	0.027
Years of T2DM>2.5 years	25,391 (51.1)	6586 (51.8)	0.015	25,454 (51.2)	6280 (50.9)	0.007
Education level						
Senior high school or higher	7251 (14.6)	1306 (10.3)	0.131	6815 (13.7)	1616 (13.1)	0.018
Junior high school	13,968 (28.1)	3572 (28.1)	0.002	13,966 (28.1)	3460 (28.1)	0.001
Primary school	16,797 (33.8)	4946 (38.9)	0.107	17,320 (34.8)	4357 (35.3)	0.011
Others	11,680 (23.5)	2880 (22.7)	0.02	11,610 (23.4)	2895 (23.5)	0.003
Sex (female)	23,501 (47.3)	6309 (49.7)	0.047	23,762 (47.8)	5975 (48.5)	0.011
Smoking	15,232 (30.7)	3892 (30.6)	0.001	15,240 (30.7)	3799 (30.8)	0.001
Drinking	17,119 (34.4)	4266 (33.6)	0.018	17,039 (34.3)	4207 (34.1)	0.002
Sport frequency						
>4 d/w	5749 (11.6)	1822 (14.3)	0.083	6038 (12.1)	1542 (12.5)	0.011
1–3 d/w	9529 (19.2)	2583 (20.3)	0.029	9651 (19.4)	2401 (19.5)	0.002
<1 d/w	34,418 (69.3)	8298 (65.3)	0.084	34,022 (68.4)	8384 (68.0)	0.009
Medication use						
Insulins	4488 (9.0)	655 (5.2)	0.151	4101 (8.2)	925 (7.5)	0.016
Metformin	13,368 (26.9)	4841 (38.1)	0.241	14,536 (29.2)	3773 (30.6)	0.028
Sulfonylureas	14,471 (29.1)	5344 (42.1)	0.273	15,800 (31.8)	4047 (32.8)	0.015
Glinides	3143 (6.3)	940 (7.4)	0.043	3255 (6.5)	813 (6.6)	0.004
Other oral antidiabetics^b^	1458 (2.9)	415 (3.3)	0.019	1500 (3.0)	395 (3.2)	0.016
PPI	6757 (13.6)	1956 (15.4)	0.051	6960 (14.0)	1768 (14.3)	0.021
ACEI	3454 (7.0)	973 (7.7)	0.027	3533 (7.1)	888 (7.2)	0.002
ARB	13,885 (27.9)	4108 (32.3)	0.096	14,340 (28.8)	3593 (29.1)	0.011
Asprin	4781 (9.6)	1105 (8.7)	0.032	4691 (9.4)	1148 (9.3)	0.012
Diuretics	7588 (15.3)	2141 (16.9)	0.043	7750 (15.6)	1910 (15.5)	0.002
Beta-blocking agents	4724 (9.5)	1141 (9.0)	0.018	4674 (9.4)	1138 (9.2)	0.001
Calcium channel blockers	14,099 (28.4)	3934 (31.0)	0.057	14,372 (28.9)	3577 (29.0)	0.005
Statins	6968 (14.0)	1987 (15.6)	0.046	7133 (14.3)	1756 (14.2)	0.002
Other lipid modifying agents	1279 (2.6)	402 (3.2)	0.035	1345 (2.7)	347 (2.8)	0.008
CCI						
0	35,642 (71.7)	9428 (74.2)	0.056	35,902 (72.2)	8980 (72.9)	0.014
1	7753 (15.6)	2065 (16.3)	0.018	7820 (15.7)	1929 (15.7)	0.002
≥2	6301 (12.7)	1211 (9.5)	0.1	5989 (12.0)	1417 (11.5)	0.017
BMI (kg/m^2^)	23.8 (2.97)	24.3 (3.00)	0.17	23.9 (2.97)	23.9 (2.94)	0.025
DBP (mmHg)	78.1 (6.02)	78.3 (6.02)	0.043	78.1 (5.97)	78.1 (5.94)	0.007
SBP (mmHg)	128.7 (9.26)	128.8 (9.10)	0.012	128.7 (9.13)	128.7 (9.07)	0.002
FPG (mmol/L)	7.3 (2.06)	7.4 (1.98)	0.005	7.3 (2.03)	7.3 (2.02)	0.003
HbA1c (%)	7.6 (1.92)	7.6 (1.87)	0.028	7.6 (1.90)	7.6 (1.90)	0.004
HDLC (mmol/L)	1.2 (0.32)	1.2 (0.31)	0.039	1.2 (0.32)	1.2 (0.31)	0.011
LDLC (mmol/L)	2.7 (0.81)	2.7 (0.81)	0.077	2.7 (0.81)	2.7 (0.81)	0.02
Outpatient visits						
0	12,183 (24.5)	2274 (17.9)	0.162	11,515 (23.2)	2818 (22.9)	0.007
1–6	19,529 (39.3)	4891 (38.5)	0.016	19,436 (39.1)	4784 (38.8)	0.006
7–12	10,150 (20.4)	3208 (25.3)	0.115	10,637 (21.4)	2656 (21.5)	0.004
>12	7834 (15.8)	2331 (18.3)	0.069	8124 (16.3)	2069 (16.8)	0.012
Inpatient admissions						
0	45,439 (91.4)	12,056 (94.9)	0.138	45,801 (92.1)	11,438 (92.8)	0.025
1	3464 (7.0)	572 (4.5)	0.106	3218 (6.5)	759 (6.2)	0.013
≥2	793 (1.6)	76 (0.6)	0.096	692 (1.4)	130 (1.1)	0.031
Year of index date						
2009	1855 (3.7)	325 (2.6)	0.067	1740 (3.5)	442 (3.6)	0.004
2010	4062 (8.2)	1127 (8.9)	0.025	4141 (8.3)	1060 (8.6)	0.01
2011	5047 (10.2)	1415 (11.1)	0.032	5144 (10.3)	1276 (10.4)	0.001
2012	5107 (10.3)	1356 (10.7)	0.013	5146 (10.4)	1275 (10.3)	0.001
2013	4126 (8.3)	1245 (9.8)	0.052	4278 (8.6)	1071 (8.7)	0.003
2014	3507 (7.1)	1176 (9.3)	0.08	3740 (7.5)	971 (7.9)	0.013
2015	3558 (7.2)	1008 (7.9)	0.029	3628 (7.3)	881 (7.1)	0.006
2016	3640 (7.3)	1039 (8.2)	0.032	3728 (7.5)	935 (7.6)	0.003
2017	4438 (8.9)	1046 (8.2)	0.025	4369 (8.8)	1087 (8.8)	0.001
2018	3857 (7.8)	733 (5.8)	0.079	3653 (7.3)	869 (7.0)	0.012
2019	3948 (7.9)	620 (4.9)	0.125	3635 (7.3)	824 (6.7)	0.025
2020	3404 (6.8)	712 (5.6)	0.052	3279 (6.6)	808 (6.6)	0.002
2021	3147 (6.3)	902 (7.1)	0.031	3230 (6.5)	827 (6.7)	0.008

*T2DM* type 2 diabetes mellitus, *TZDs* thiazolidinediones, *AGIs* alpha glucosidase inhibitors, *ACEI* angiotensin-converting enzyme inhibitors, *ARB* angiotensin receptor blockers, *PPI* proton-pump inhibitors, *CCI* Charlson comorbidity index, *BMI* body mass index, *FPG* fast plasma glucose, *HbA1c* glycated hemoglobin, *HDLC* high-density lipoprotein cholesterol, *LDLC* low-density lipoprotein cholesterol, *SBP* systolic blood pressure, *DBP* diastolic blood pressure.

^a^For continuous variables, the values are mean (standard deviation); for categorical variables the values are number (percentage).

^b^These drugs included dipeptidyl peptidase 4 (DPP-4) inhibitors and sodium-glucose co-transporter 2 (SGLT2) inhibitors.

### Association between TZD use and PD incidence

**Fig. 2:**
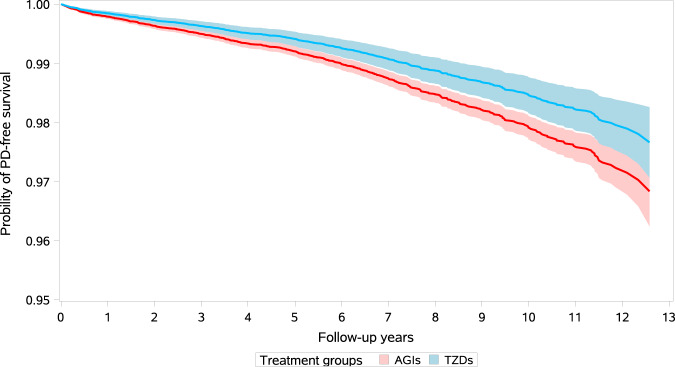
IPTW-weighted survival curves of TZD users and AGI users. AGIs alpha glucosidase inhibitors, TZDs thiazolidinediones.

A total of 674 incident cases of PD were observed during follow-up in the primary analysis, of them 571 were AGI users and 103 were TZD users, with an incidence of 203 and 135 per 100,000 person-years, respectively. Compared with users of AGIs, the crude analysis indicated a significant inverse association between use of TZDs and incidence of PD, with a hazard ratio (HR) of 0.67 (95% confidence interval [CI], 0.54–0.82) (Table 2). After adjusting for potential confounders using stabilized inverse probability of treatment weighting (IPTW), users of TZDs still showed a significant 26% decrease in incidence of PD (HR 0.74; 95% CI, 0.59–0.92). The mean of the IPTW was 1.00 (standard deviation, 0.24), and the median was 0.97 (IQR, 0.50–1.05). Multivariate regression model presented consistent result, with a HR of 0.76 (95% CI, 0.62–0.95). Figure 2 gives the IPTW-weighted survival curves of the users of AGIs and TZDs. Moreover, subgroup analyses found similar inverse association between use of TZDs and incidence of PD in different subpopulations of T2DM patients except that in smokers TZD use showed an insignificant increase of risk of PD (HR 1.02; 95% CI, 0.64–1.64). No significant interaction was indicated in all subgroup analyses (Table 2). In addition, the IPTW adjusted HRs for the cumulative duration of TZD use of ≤0.5 years, 0.51–4.0 years, and >4 years were 0.80 (95% CI, 0.51–1.26), 0.69 (95% CI, 0.45–1.07), and 0.73 (95% CI, 0.55–0.98), respectively (Supplementary Table 1).

**Table 2:** Results of the primary and subgroup analyses.

	AGI users		TZD users		HR (95% CI)		
	Cases/person-years	Incidence (/100,000 PY)	Cases/person-years	Incidence (/100,000 PY)	Crude analysis	Multivariate regression	IPTW model
Overall	571/281,268	203.0	103/76,275	135.0	0.67 (0.54–0.82)	0.76 (0.62–0.95)	0.74 (0.59–0.92)
Age group							
≤60 years	139/134,268	103.5	27/41,284	65.4	0.64 (0.42–0.96)	0.65 (0.43–0.99)	0.60 (0.39–0.92)
>60 years	432/147,001	293.9	76/34,992	217.2	0.74 (0.58–0.95)	0.83 (0.64–1.06)	0.80 (0.62–1.04)
Sex							
Male	294/142,197	206.8	52/37,249	139.6	0.68 (0.50–0.91)	0.83 (0.61–1.13)	0.76 (0.56–1.04)
Female	277/139,071	199.2	51/39,027	130.7	0.66 (0.49–0.89)	0.71 (0.52–0.96)	0.71 (0.51–0.98)
Years of T2DM							
≤2.5 years	246/138,840	177.2	48/38,494	124.7	0.71 (0.52–0.96)	0.80 (0.58–1.10)	0.78 (0.56–1.08)
>2.5 years	325/142,428	228.2	55/37,781	145.6	0.64 (0.48–0.86)	0.74 (0.55–0.99)	0.71 (0.52–0.97)
Drinking							
No	373/184,040	202.7	68/50,716	134.1	0.66 (0.51–0.86)	0.75 (0.57–0.98)	0.73 (0.55–0.97)
Yes	198/97,228	203.6	35/25,559	136.9	0.68 (0.47–0.98)	0.82 (0.55–1.20)	0.75 (0.50–1.12)
Smoking							
No	455/199,268	228.3	76/54,144	140.4	0.62 (0.48–0.79)	0.71 (0.55–0.92)	0.66 (0.50–0.86)
Yes	116/82,000	141.5	27/22,132	122.0	0.87 (0.56–1.35)	1.00 (0.63–1.58)	1.02 (0.64–1.64)
BMI							
≤24 kg/m^2^	321/154,296	208	52/36,718	141.6	0.68 (0.48–0.96)	0.76 (0.54–1.08)	0.73 (0.51–1.05)
>24 kg/m^2^	250/126,972	196.9	51/39,557	128.9	0.66 (0.46–0.94)	0.77 (0.55–1.10)	0.74 (0.50–1.09)
CCI							
0	402/220,316	182.5	72/61,003	118.0	0.65 (0.50–0.83)	0.75 (0.58–0.96)	0.74 (0.56–0.96)
>0	169/60,952	277.3	31/15,272	203.0	0.75 (0.51–1.10)	0.84 (0.56–1.24)	0.75 (0.50–1.13)
FPG							
≤7 mmol/L	320/150,025	213.3	64/39,888	160.5	0.75 (0.55–1.02)	0.86 (0.63–1.18)	0.84 (0.60–1.17)
>7 mmol/L	251/131,244	191.2	39/36,388	107.2	0.56 (0.38–0.85)	0.65 (0.44–0.97)	0.60 (0.39–0.92)
HbA1c							
≤7%	245/112,805	217.2	43/30,800	139.6	0.64 (0.39–1.05)	0.73 (0.45–1.18)	0.73 (0.47–1.11)
>7%	326/168,463	193.5	60/45,475	131.9	0.69 (0.47–1.01)	0.79 (0.55–1.14)	0.75 (0.53–1.05)

*AGIs* alpha glucosidase inhibitors, *TZDs* thiazolidinediones, *FPG* fasting plasma glucose, *HbA1c* glycated hemoglobin, *BMI* body mass index, *PY* person-years, *IPTW* inverse probability of treatment weighting.

### Sensitivity analyses

Table 3 shows the results of per-protocol (PP) analysis and all sensitivity analyses. In the PP analysis, participants were censored when ceasing the initial drug, resulting in a mean follow-up of 2.2 years. Crude and IPTW analyses showed a HR of 0.65 (95% CI, 0.44–0.95) and 0.81 (95% CI, 0.53–1.21), respectively. When further adjusting for potential selection bias due to artificial censoring using inverse probability of censoring weighting (IPCW), the HR changed to 0.73 (95% CI, 0.45–1.20).

**Table 3:** Results of per-protocol and sensitivity analyses.

	AGI users		TZD users		HR (95% CI)
	Cases/person-years	Incidence (/100,000 PY)	Cases/person-years	Incidence (/100,000 PY)	
Per-protocol analyses					
Crude analysis	222/111,760	198.6	29/22,666	127.9	0.65 (0.44–0.95)
IPTW model	222/111,760	198.6	29/22,666	127.9	0.81 (0.53–1.21)
IPTW and IPCW model	222/111,760	198.6	29/22,666	127.9	0.73 (0.45–1.20)
Alternative washout periods					
12 months	538/267,478	201.1	102/73,904	138.0	0.76 (0.60–0.95)
18 months	500/252,231	198.2	99/71,311	138.8	0.77 (0.61–0.97)
24 months	469/237,329	197.6	92/67,928	135.4	0.76 (0.59–0.96)
Excluding potential secondary PD	566/281,258	201.2	103/76,275	135.0	0.74 (0.59–0.93)
Alternative weighting model					
Untruncated IPTW	571/281,268	203.0	103/76,275	135.0	0.78 (0.61–1.00)
Unstabilized IPTW	571/281,268	203.0	103/76,275	135.0	0.74 (0.59–0.92)
Competing risk model	571/281,268	203.0	103/76,275	135.0	0.74 (0.60–0.91)
Subpopulation aged ≥40 years	566/270,742	209.1	103/72,902	141.3	0.74 (0.59–0.93)
Alternative outcome definitions^a^					
Outcome definition 2	551/281,268	195.9	101/76,275	132.4	0.75 (0.60–0.94)
Outcome definition 3	455/281,268	161.8	86/76,275	112.7	0.77 (0.60–0.99)
Outcome definition 4	680/281,268	241.8	122/76,275	159.9	0.74 (0.60–0.90)
Monotony therapy of TZDs or AGIs	316/154,187	204.9	42/34,039	123.4	0.73 (0.53–1.00)

*AGI* alpha glucosidase inhibitors, *TZD* thiazolidinediones, *IPTW* inverse probability of treatment weighting, *IPCW* inverse probability of censoring weighting, *PD* Parkinson’s disease.

^a^Outcome definition 2: Parkinson’s disease defined as having at least two diagnoses of G20; Outcome definition 3: Parkinson’s disease defined as having over two diagnoses of G20 and prescriptions of anti-Parkinson agents (ATC N04) after the first diagnosis; Outcome definition 4: Parkinson’s disease defined as having a consecutive diagnosis of G20 or prescription of anti-Parkinson agents within 1 year of the first diagnosis of PD.

**Fig. 3:**
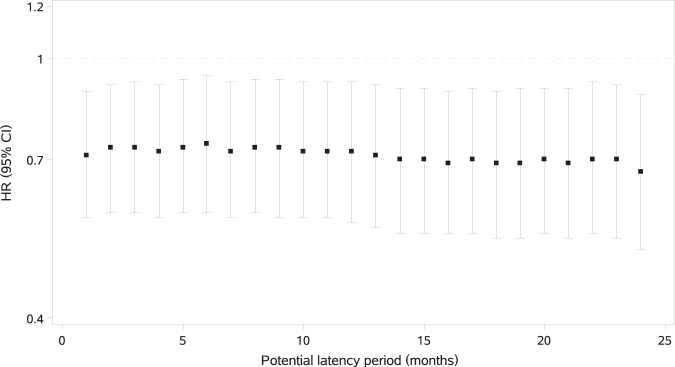
Sensitivity analyses for different potential latency periods. The length of latency period increased by one month in each sensitivity analysis.

All sensitivity analyses were consistent with that of the primary analysis (Table 3). First, analysis using a washout period of 1 year indicated that the HR of TZD use was 0.76 (95% CI, 0.60–0.95). Further results of washout periods of 18 and 24 months were consistent. Then the second sensitivity analysis got a HR of 0.74 (95% CI, 0.59–0.93) after excluding five cases of potential secondary PD. Third, when the IPTW was not truncated, analysis presented a HR of 0.78 (95% CI, 0.61–1.00), indicating extreme weights might bias the result to the null value. In contrast, result of the unstabilized IPTW model was the same as the primary analysis. Fourth, competing risk due to all-cause mortality did not appear to an issue as the subdistribution hazard model yielded a HR of 0.74 (95% CI, 0.60–0.91), almost the same as that in the primary analysis. Fifth, when we restricted the population to T2DM patients aged 40 years or older, a HR of 0.74 (95% CI, 0.59–0.93) was observed. Sixth, results of different definitions of incident PD were consistent, with HRs ranged from 0.74 to 0.77. Seventh, in the subcohort of participants who initiated monotony therapy of TZDs, we got a HR of 0.73 (95% CI, 0.53–1.00) compared with monotony treatment of AGIs. Finally, excluding potential latency periods of different length resulted in similar HR estimates, all of which were significant lower than 1, ranging from 0.67 (95% CI, 0.51–0.88) to 0.74 (95% CI, 0.58–0.94) (Fig. 3).

## Discussion

In this long-term population-based cohort study, participants were followed-up for a maximum of approximating 13 years and we found that use of TZDs was associated with a 26% decrease in the incidence of PD compared with AGI use in patients with T2DM. Results of an emulation of PP effects in which follow-up was censored when participants discontinuing the initial treatment, showed a HR in the same direction and of similar magnitude as those observed in primary analyses. Further, our results were consistent in various subgroup analyses and robust across different sensitivity analyses.

To the best of our knowledge, no previous cohort study has investigated the association between TZD use and risk of PD in a mainland Chinese population. PD is the fastest growing neurological disorder^1,10^, causing a substantial amount of disease burden worldwide. This disease is a particular concern in China, where an estimate of 3.6 million people aged 60 years or above live with PD^11^, accounting for 23% of the entire global PD population^1,12^. Further, China has the fastest increasing in prevalence of PD, which was more than five folds that of the global average^10,12^. It is estimated that the number of PD patients in China will increase to around five million by 2030, accounting for 60% of patients with PD of the whole world^10^. Therefore, our findings of the potential neuroprotective effect of TZDs in T2DM patients might provide some insights into the development of effective prevention and control measures to reduce the future burden of PD^10,11^.

Our findings were consistent with previous studies in vivo and in vitro. PD pathogenesis involves multiple mechanisms, among which in vivo and in vitro studies have shown that TZDs may play a role in improving mitochondrial function and reducing insulin resistance, oxidative stress, and inflammation^4,13^. TZDs are a kind of agonist of peroxisome proliferator-activated receptor γ (PPARγ), reducing insulin resistance through regulating genes involving insulin sensitivity^4,7^. They can also bind to the outer mitochondrial membrane protein (mitoNEET), which plays a key role in electron transport and oxidative phosphorylation, to regulate neuronal complex I activity in neuronal cells, reducing oxidative stress and cell death^4,7^. Moreover, animal experiments have shown that pioglitazone is able to protect against lipopolysaccharide-mediated inflammation and dopaminergic neurodegeneration in the rat brain^14^. Also, TZDs present neuroprotective effects in 1-methyl-4-phenyl-1,2,3,6-tetrahydropyridine (MPTP) treated mice through influencing attenuation of microglial response, activation of kappa-light-chain-enhancer of activated B-cells (NF-κB) and production of inflammatory cytokines^15,16^.

Despite showing promise in multiple researches of the potential mechanisms through which TZDs may have effects on PD, only a limited number of clinical studies have investigated the association between TZD use and PD incidence in diabetic patients. Among these, the findings of this study were consistent with several previous studies conducted in the United Kingdom^17^, Norway^18^, and Taiwan region^19,20^, of which all had a follow-up time of more than 5 years, and reported a significant reduction in the incidence of PD in TZD users, with a HR ranging from 0.40 to 0.72. No association between TZD use and risk of PD was observed in other studies^6,18,21^. However, all of these studies followed participants for an average of less than 5 years, which may not long enough to observe the protective effect of TZDs^8,9^, since PD has a long onset period^22^. This was also reflected in our PP analysis, in which participants were censored at cessation of initial treatment and thus resulted in an average of 2 years of follow-up. The PP analysis observed an insignificant but similar magnitude of reduction in PD incidence in TZD users as that in primary analysis. In addition, a case-control study in Finland reported that TZD use might reduce PD risk by 22%^22^, approximating our results. Furthermore, several methodological issues should be noted in previous studies. Various comparison groups have been used in previous studies, among which the majority used non-users of TZDs as the control^19–21,23,24^. Some studies even included patients who did not receive any glucose lowering drugs^19–21,23^. This could induce several biases in the results, including confounding by indication and immortal time bias^25,26^. Since TZDs are usually prescribed as a second-line drug, studies included patients only receiving treatment of metformin which is the first-line choice for glycemic control in the comparison group^18–21,23^, had a risk of time-lag bias^26^. Another limitation that most studies had was the uncontrolled confounding by lifestyle factors, such as smoking and drinking status, and disease severity of diabetes, which has been shown to be associated with the risk of PD^2,8^.

In this study we applied an active-comparator new-user (ACNU) design using AGIs which were another class of commonly used second-line hypoglycemic drugs in the study population^27^ as the comparator, with a rich set of potential confounders, including lifestyle factors and disease severity measures, thus mitigating time-related bias and confounding by indication^25,28^. However, despite these epidemiological studies, clinical trials investigating effects of TZDs on PD risk are scarce. A phase 2 randomized controlled trial concluded that pioglitazone had no effects on modifying progression in early PD^29^. Nevertheless, this trial only included early PD patients without diabetes. Therefore, the negative findings might be due to the exclusion of patients with diabetes, as diabetes and PD share some common pathogenesis^9^. Therefore, TZDs may only have a neuroprotective effect in diabetes patients, but not in nondiabetes population^8,9^. Furthermore, a short follow-up of 44 weeks in this trial might not be sufficient to observe a significant protective effect with clinical value^8,9^. In contrast, our study was aimed to assess the association between TZD use and PD incidence and could not determine whether this drug could modify the progression of PD after disease onset. Furthermore, our results did not indicate any significant effect modification by other risk factors in the association between TZD use and PD risk. This was consistent with several previous studies, which also found no interaction between TZD use and age or sex^6,21^. However, potential heterogeneity of treatment effects should be noted in some population, as our results indicated that TZD use might have a stronger effect in T2DM patients under 60 years of age. Further studies with larger sample size are need for investigating potential heterogeneous effects of TZD use on PD risk.

Several limitations should be noted in this study. First, participants in this study were from a single municipal district in China, thus caution should be exercised when extrapolating the findings of this study to other populations. Second, although we considered quite a number of time-invariant and time-varying covariates in the analyses and ACNU design might have further mitigated the bias related to unmeasured confounding^25^, some potential confounders, such as dietary patterns, were not adjusted due to lack of accurate information in the database. Third, the database did not record complete dosage information of drugs, making it difficult to study the relationship between cumulative TZD exposure and PD incidence. Our analyses of cumulative duration of TZDs might not accurately reflect the cumulative exposure dose of this drug, thus more studies focusing on the dose-response relationship between cumulative TZD exposure and risk of PD would provide further valuable evidence. Finally, like previous studies^6,18–21,24^ we used a by-proxy definition of PD. The PD incidence in our primary analysis was higher than that in diabetic patients in Western countries^6,18,24^, but similar to that in the Korea^23^. To adjust for potential bias caused by misclassification of PD cases, we applied several different outcome definitions combing diagnosis and prescription records related to PD. Further, we excluded potential secondary PD. Sensitivity analyses of various PD definitions were highly consistent, thus suggesting that there might be little differences in misclassification of outcome between users of AGIs and TZDs and our results were robust against potential misclassification bias.

In conclusion, we found a potential protective effect of TZD use against PD incidence in a Chinese T2DM population. Given the heavy disease burden of diabetes and PD in China, our results can provide some evidence for selection of oral glucose lowering agents for T2DM in clinical practice.

## Methods

### Data source

Participants were drawn from the Yinzhou Regional Health Care Database (YRHCD), which integrated longitudinal information of electronic medical records, disease registry and management, death registry and other healthcare services in the Yinzhou District, Ningbo City of China^27,30^. In 2008, disease registry and management systems were established for diabetes mellitus, cancer, cardiovascular disease, hypertension, and chronic obstructive pulmonary disease. Diabetes patients were registered in the disease surveillance system once diagnosed and would be followed up at least four times a year by community physicians, with common health measures, including blood pressure, fasting plasma glucose (FPG), glycated hemoglobin (HbA1c) being measured or asked^27^. In this study, disease registry system and electronic diagnosis records were linked and patients with T2DM were included if they: (1) were registered in the diabetes registry system and diagnosed with T2DM; or (2) had more than two diagnosis records of T2DM and no records of type 1 diabetes in the electronic medical records. Longitudinal records of drug prescription, laboratory examination, and outpatient and inpatient visits were linked for information of drug exposure, covariates, and outcome capture. The data used in this study and their relationship were presented in the Supplementary Fig. 1.

### Exposure and cohort

We applied an ACNU design, using a comparator of alpha glucosidase inhibitors (AGIs), which are another class of second-line oral glucose lowering agents commonly used at the same stage of T2DM as TZDs in China^31^. Cohort of T2DM patients who were new users of TZDs or AGIs after January 1, 2009 were assembled. Drug prescriptions were identified by classification code of Anatomical Therapeutic Chemical (ATC) system. TZDs (ATC A10BG) used in the study population contained pioglitazone and rosiglitazone, and AGIs (ATC A10BF) included acarbose, miglitol, and voglibose (Supplementary Table 2). New users were identified by using a baseline washout period of 6 months before the first fill of TZDs or AGIs, during which participants could not receive prescription of a drug from either class. The date of the first fill was defined as the index date.

We excluded participants who were younger than 18 years and who initiated combination treatment of TZDs and AGIs at the index date, and who had received a diagnosis of PD before the index date. We further excluded patients who did not take at least two consecutive prescriptions of TZDs or AGIs within 6 months of the index date to ensure that participants actually started on these drugs.

The study was approved by the ethical review board of Peking University Health Science Center (approval number: IRB00001052-18013-Exempt). Informed consent was not required owing to the use of anonymized routine data.

### Outcome and follow-up

The primary outcome was the incident diagnosis of PD, which was defined as (1) having at least two consecutive diagnosis code or description of G20 from the International Classification of Diseases, 10th Revision (ICD-10), or (2) having at least one diagnosis record of PD and prescription records of anti-Parkinson agents (ATC N04, Supplementary Table 3). The date of the first diagnosis was defined as the outcome date.

Our primary analysis was to assess the any-exposure intention-to-treat (ITT) effects of TZD use on the risk of PD. Thus, participants were followed-up from the index date until the first occurrence of the following events: diagnosis of PD, death, last medical record in the database, or the end of the study period (December 31, 2021).

### Covariates

Covariates were measured in the baseline washout period and included demographic characteristics (age, sex, and education level); behavior and lifestyle (smoking, drinking, and regular exercise); duration of T2DM; comorbidities measured as CCI (Supplementary Table 4)^32^; co-use of prescription drugs, including other antidiabetic drugs except TZDs and AGIs (insulins, metformin, sulfonylureas, glinides, and other oral glucose lowering agents), common medications for cardiovascular diseases (diuretics, beta-blocking agents, calcium channel blockers, angiotensin-converting enzyme inhibitors (ACEI), angiotensin receptor blockers (ARB), and aspirin), lipid modifying agents, and proton-pump inhibitors (PPI). Further, blood glucose level (FPG and HbA1c), blood lipid level, blood pressure, body mass index (BMI), and healthcare utilization (inpatient and outpatient visits) were also included.

### Statistical analyses

Descriptive statistics (mean and standard deviation for continuous covariates and frequency and percentage for categorical variables) summarized baseline covariates and standardized mean difference (SMD) was used for comparisons between initiators of TZDs and AGIs as suggested by Austin et al.^33^. A SMD less than 0.1 was used to show comparable balance in the covariates^33^. Inverse probability of treatment weighting (IPTW) was applied for controlling baseline confounding and a Cox regression model was used to estimate the HR with 95% confidence interval (CI) for the association between TZD use and PD. We fitted a logistic regression model to estimate the stabilized IPTW for each subject. The denominator of the IPTW was the probability of receiving the actual drug treatment condition on all measured covariates listed above. Continuous variables such as FPG, HbA1c were modeled as a restricted cubic spline with knots at 5th, 25th, 50th, 75th, and 95th percentiles. The numerator of the IPTW was the marginal probability of TZD or AGI use in the overall sample. We also included the year of index date in the model for IPTW to adjust for changes in prescribing patterns over time. The final stabilized IPTW was truncated at the 1st/99th percentile for mitigating impacts of extreme weights. Another two standard Cox models with different confounding adjustment strategies were provided for comparison: unadjusted and multivariate regression adjusted. Multiple imputation was applied for imputing missing data using the full conditional specification method with five imputations according to the quadratic rule recommended by von Hippel^34^. The proportional hazards assumption was tested using the Schoenfeld residuals method and no violation of this assumption was found. Robust variance was used for calculating the 95% CIs of HRs when applying weighted models.

### Subgroup analyses and cumulative duration of TZD use

We examined the association of TZD use and incidence of PD within different subgroups for checking potential interactions between the TZD use and baseline characteristics: age (≤60 and >60 years), sex (female and male), CCI (0 and ≥1), smoking and drinking behavior, FPG (≤7 mmol/L and >7 mmol/L), HbA1c (≤7% and >7%), BMI (≤24 kg/m^2^ and >24 kg/m^2^), and duration of diabetes at the index date (≤2.5 years and >2.5 years).

Cumulative duration of TZD use was calculated as the period between the index date and the date of the last TZD prescription. Tertiles of cumulative duration were used to define a categorical cumulative TZD therapy. We then examined the association between different duration (≤0.5, 0.51–4, and >4 years) of TZD treatment and PD incidence compared with use of AGIs.

### Sensitivity analyses

We emulated a per-protocol (PP) analysis to examine the effects of sustained exposure, in which participants were further censored upon discontinuation of the initial drug in addition to the censoring reasons in the ITT analyses. Treatment discontinuation was defined as no further refill of the initial drug within 6 months of the previous prescription. This artificial censoring could induce selection bias and could be influenced by time-varying confounders^35^. Thus, we applied a marginal structural model with time-varying inverse probability of censoring weighting (IPCW) to adjust for selection bias introduced by artificial censoring. IPCW was the inverse of the probability of remaining uncensored at each follow-up conditioned on time-invariant and time-varying factors, and was estimated using a pooled logistic model. Time-varying factors were all covariates listed above except demographic characteristics, behavior and lifestyle, duration of T2DM, and year of index date. We used a 6-month interval for assessing time-updated exposure and covariates at the beginning of each new period in the PP analysis.

We further performed multiple sensitivity analyses to examine the robustness of the results in our primary analysis. First, alternative washout periods of 12, 18, and 24 months were applied for defining new users of TZDs and AGIs. Second, we excluded all possible cases of secondary PD, who had a diagnosis of ICD-10 code G21 or G22 after receiving first diagnosis of G20. Third, unstabilized and untruncated stabilized IPTW were applied. Forth, the Fine-Gray subdistribution hazard model was used to check possible competing risk by death from any cause. Fifth, we restricted the study population to T2DM patients who aged over 40 years at the index date. This could help exclude cases of early-onset PD since young people rarely develop this disease. Sixth, we used several alternative definitions of incident PD as: (1) having at least two diagnoses of G20; (2) having over two diagnoses of G20 and prescriptions of anti-Parkinson agents (ATC N04) after the first diagnosis; and (3) having a consecutive diagnosis of G20 or prescription of anti-Parkinson agents within one year of the first diagnosis of PD. Seventh, we excluded T2DM patients who received any antidiabetic drugs in the baseline washout period and evaluated the effects of monotony therapy of TZDs compared with monotony treatment of AGIs. Finally, we excluded a period after the index date for adjusting potential latency time. For example, we identified participants with over 6 months of use of the initial drug and follow-up were begun 6 months after the index date. This analysis was repeated with requirements of successively longer minim time of follow-up by adding 1 month to each analysis, up to a maximum of 24 months. This kind of analysis could also help adjust for unmeasured confounding by undiagnosed disease^35^.

Statistical analysis was performed using SAS 9.4 (SAS Institute Inc., Cary, NC, USA). All statistical tests were conducted two-sided and a *p*-value < 0.05 was considered to indicate statistical significance

## Supplementary information

Supplementary materials

## Data Availability

YRHCD is open for research purposes. Data requests should be sent to the Yinzhou District Center for Disease Control and Prevention, by contacting Mr. Peng Shen at shen-peng@foxmail.com or Mr. Yexiang Sun at 19464337@qq.com. The feasibility, novelty, and scientific rigor of the proposal will be discussed, and the decision whether to share the data will be made by an academic committee. The data that support the findings of this study are available from the corresponding author upon reasonable request.

## Code availability

The code for this study is available in a ResearchGate repository (https://www.researchgate.net/publication/363771107_Code_TZD_PD).
