# Supplementary material for: Thiazolidinedione use and risk of Parkinson’s disease in patients with type 2 diabetes mellitus
Source: NPJ Parkinsons Dis. 2022 Oct 21;8:138. doi: 10.1038/s41531-022-00406-8 (PMC9587207; doi:10.1038/s41531-022-00406-8)
Supplement: Supplementary file 1 — Supplementary materials [file 41531_2022_406_MOESM1_ESM.pdf]

Table of Contents

Supplementary Table 1. Association between incidence of Parkinson's disease and cumulative duration of TZD use 2

Supplementary Fig. 1: Data structure of the YRHCD. 3

Supplementary Table 2. Alpha glucosidase inhibitors and thiazolidinediones used in the study population 4

Supplementary Table 3. Anti-Parkinson drugs used in the study population 5

Supplementary Table 4. Comorbidities used to define Charlson comorbidity index 6

Supplementary Table 1. Association between incidence of Parkinson's disease and cumulative duration of TZD use

	Cases/Person years	Incidence (/100000 PY)	HR (95% CI)
**AGI use**	571/281268	203.0	Reference
**Cumulative TZD use**			
**Crude analysis**			
≤0.5 years	22/15476	142.2	0.72 (0.47–1.10)
≤4 years	25/20752	120.5	0.62 (0.41–0.92)
>4 years	56/40048	139.8	0.67 (0.51–0.88)
**Multivariate regression**			
≤0.5 years	22/15476	142.2	0.81 (0.53–1.25)
≤4 years	25/20752	120.5	0.69 (0.46–1.04)
>4 years	56/40048	139.8	0.78 (0.59–1.03)
**IPTW model**			
≤0.5 years	22/15476	142.2	0.80 (0.51–1.26)
≤4 years	25/20752	120.5	0.69 (0.45–1.07)
>4 years	56/40048	139.8	0.73 (0.55–0.98)

Abbreviations: PY: person-years; T2DM: type 2 diabetes mellitus; TZDs: thiazolidinediones; AGIs: alpha glucosidase inhibitors; IPTW: inverse probability of treatment weighting.

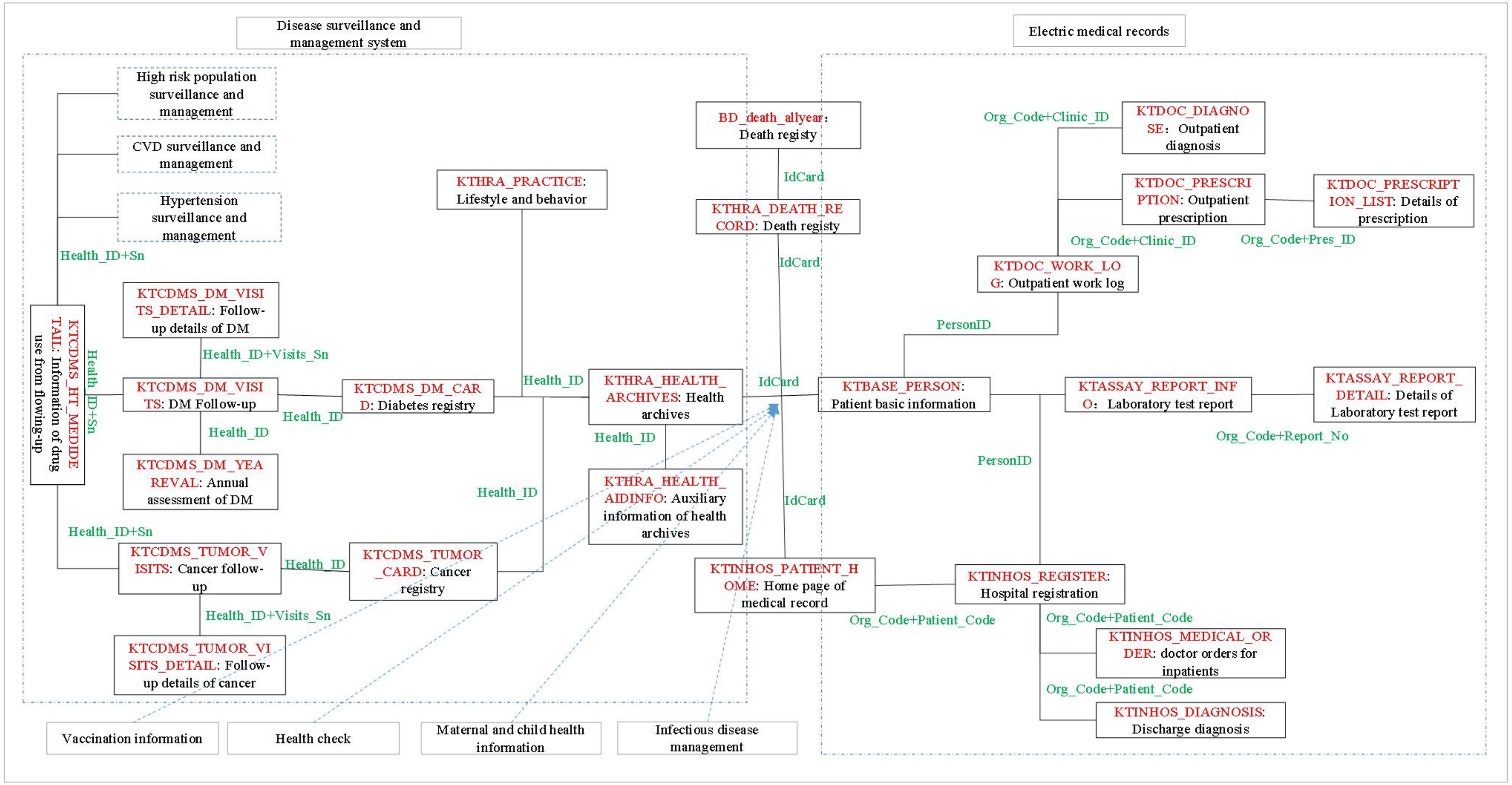

Supplementary Fig. 1: Data structure of the YRHCD.

Relationship of different tables is presented in the figure. Data from the disease surveillance and management and electric medical records were used in this study. Detailed information contained in each table in the figure and more details of other data can be found elsewhere[1, 2].

Red words are table names. Green words are keys for data linking.

Supplementary Table 2. Alpha glucosidase inhibitors and thiazolidinediones used in the study population

Alpha glucosidase inhibitors			Thiazolidinediones	
ATC code	Drug name		ATC code	Drug name
A10BF01	Acarbose		A10BG03	Pioglitazone
A10BF02	Miglitol		A10BG02	Rosiglitazone
A10BF03	Voglibose		A10BD05	Metformin and pioglitazone
A10BD17	Metformin and acarbose		A10BD03	Metformin and rosiglitazone

Supplementary Table 3. Anti-Parkinson drugs used in the study population*

ATC code	Drug name
N04AA01	Trihexyphenidyl
N04BA01	Levodopa
N04BA02	Levodopa and decarboxylase inhibitor
N04BA03	Levodopa, decarboxylase inhibitor and COMT inhibitor
N04BB01	Amantadine
N04BC01	Bromocriptine
N04BC02	Pergolide
N04BC05	Pramipexole
N04BC08	Piribedil
N04BC09	Rotigotine
N04BD01	Selegiline
N04BD02	Rasagiline
N04BX02	Entacapone

*Only drugs of N04 prescribed in the study population are included in this table.

Supplementary Table 4. Comorbidities used to define Charlson comorbidity index

Comorbidities[a]	Defined according to ICD-10 codes.	Adjustment in the model
Myocardial infarction	I21.x, I22.x, I25.2, I25.5	For calculating CCI, not included in the final analysis models
Congestive heart failure	I11.0, I13.0, I13.2, I50	Ditto
Peripheral vascular disease	I70, I71, I73.1, I73.8, I73.9, I77.1, I79.0, I792, K55.1, K55.8, K55.9, Z95.8, Z95.9	Ditto
Cerebrovascular disease	G45, G46, H34.0, I60-I69	Ditto
Chronic pulmonary disease	I278, I279, J40-J47, J60-J67, J684, J701, J703	Ditto
Connective tissue disease	M05, M06, M31.5, M32, M33, M34, M35.1, M353, M36.0	Ditto
Ulcer disease	K25-K28	Ditto
Mild liver disease	B18, K70.0-K70.3, K709, K71.3-K71.5, K71.7, K73, K74, K76.0, K76.2-K76.4, K76.8, K76.9, Z94.4	Ditto
Diabetic complication	E10.2-E10.5, E10.7, E11.2-E11.5, E11.7, E12.2-E12.5, E12.7, E13.2-E13.5, E13.7, E14.2-E14.5, E14.7	Ditto
Hemiplegia	G041, G114, G801, G802, G81, G82, G830, G831-G834, G839	Ditto
Diabetes with chronic complication	E102-E105, E107, E112-E115, E117, E122-E125, E127, E132-E135, E137, E142-E145, E147	Ditto
Moderate or severe renal disease	I120, I131, N032-N037, N052-N057, N18, N19, N250, Z49.0-Z49.2, Z940, Z992	Ditto
Any tumor (including lymphoma and leukemia except for malignant neoplasm of skin)	C00-C26, C30-C34, C37-C41, C43, C45-C58, C60-C76, C81-C85, C88, C90-C97	Ditto
Moderate or severe liver disease	I850, I859, I864, I982, K704, K711, K721, K729, K765-K767	Ditto
Metastatic solid tumor	C77-C80	Ditto
HIV/AIDS	B20-B22, B24, Z21	Ditto

[a] Defined according to the 10th version of International Classification of Disease (ICD-10).

References

1. Lin H, Tang X, Shen P, et al. Using big data to improve cardiovascular care and outcomes in China: a protocol for the CHinese Electronic health Records Research in Yinzhou (CHERRY) Study. *BMJ Open*. 2018;8(2):e19698.

2. Zhao H, Liu Z, Zhuo L, et al. Sulfonylurea and cancer risk among patients with type 2 diabetes: a population-based cohort study. *Front Endocrinol.* 2022;13.
